# Menkes disease complicated by concurrent ACY1 deficiency: A case report

**DOI:** 10.3389/fgene.2023.1077625

**Published:** 2023-03-02

**Authors:** Alessia Mauri, Laura Assunta Saielli, Enrico Alfei, Maria Iascone, Daniela Marchetti, Elisa Cattaneo, Anna Di Lauro, Laura Antonelli, Luisella Alberti, Eleonora Bonaventura, Pierangelo Veggiotti, Luigina Spaccini, Cristina Cereda

**Affiliations:** ^1^ Department of Biomedical and Clinical Sciences, University of Milan, Milan, Italy; ^2^ Center of Functional Genomics and Rare Diseases, Buzzi Children’s Hospital, Milan, Italy; ^3^ Pediatric Neurology Unit, Buzzi Children’s Hospital, Milan, Italy; ^4^ Medical Genetics Laboratory, Bergamo, Italy; ^5^ Clinical Genetics Unit, Buzzi Children’s Hospital, Milan, Italy

**Keywords:** case report, Menkes disease, novel ATP7A variant, ACY deficiency, overlapping phenotype, delayed copper therapy

## Abstract

**Introduction:** Menkes disease is an X‐linked recessive condition caused by mutations in the *ATP7A* gene, which leads to severe copper deficiency. Aminoacylase-1 deficiency is a rare inborn error of metabolism caused by homozygous or compound heterozygous variant in the *ACY1* gene, characterized by increased urinary excretion of specific N-acetyl amino acids.

**Case presentation:** We report an infant with neurological findings such as seizures, neurodevelopmental delay and hypotonia. Metabolic screening showed low serum copper and ceruloplasmin, and increased urinary excretion of several N-acetylated amino acids. Whole-exome sequencing analysis (WES) revealed the novel *de novo* variant c.3642_3649dup (p.Ala1217Aspfs*2) in the *ATP7A* gene, leading to a diagnosis of Menkes disease, and the simultaneous presence of the homozygous *ACY1* variant c.1057C>T (p.Arg353Cys) causative of Aminoacylase-1 deficiency.

**Conclusion:** Our patient had two rare conditions with different treatment courses but overlapping clinical features. The identified novel *ATP7A* mutation associated with Menkes disease expands the *ATP7A* gene spectrum.

## Introduction

Menkes disease (MNK; #309400) is an X-linked recessive condition that occurs in 1 in 100,000–300,000 live births. It is caused by hemizygous variants in male or heterozygous variants in female with skewed X inactivation in the *ATP7A* gene (Xq21.1; #300011) (Caicedo-Herrera et al., 2018). To date, more than 400 different pathogenic variants affecting this gene have been reported [Leiden Open Variation Database v3.0 (www.LOVD.nl/ATP7A)]. The type of reported mutations found includes chromosome aberrations, point mutations, small and gross deletion/insertions (De Gemmis et al., 2017; Kaler et al., 2020).

The *ATP7A* gene encodes a copper-transporting ATPase involved in two important cellular functions: to facilitate the copper transport across membranes from non-hepatic tissues and to deliver copper to the secretory pathway for incorporation into copper-dependent enzymes (Chen et al., 2020). A genetic defect in this transmembrane protein would lead to severe copper accumulation in some tissues, such as the small intestine and kidneys, while some tissues, including the brain, have unusually low levels. The decreased supply of copper can reduce the activity of numerous copper-containing enzymes, affecting the structure and function of the nervous system, bones, skin, hair, and blood vessels (Horn and Wittung-Stafshede, 2021).

Menkes clinical features include growth retardation, bony abnormalities, neurodevelopmental delays, hypotonia, seizures and unusual “kinky” hair. Daily subcutaneous administration of copper histidinate is the only current treatment and has been associated with variable outcomes depending on the age of initiation and *ATP7A* mutation type. Without treatment, premature death is typical, often by age 3 years (Kaler et al., 2003; Altarelli et al., 2019).

Aminoacylase-1 deficiency (ACYD; #609924) is an inborn error of metabolism characterized by increased urinary excretion of N-acetylated amino acids (Sass et al., 2006). Phenotypes reported in association with ACYD vary significantly including normal clinical findings and a variety of neurological features such as intellectual disability, motor delay, seizures, moderate to severe intellectual disability, absent speech, growth delay, spine malformations, muscle weakness, hypotonia, and autistic features (Alessandrì et al., 2018).

ACYD is caused by homozygous or compound heterozygous mutations in the Aminoacylase-1 gene (*ACY1*, 3p21.2). The *ACY1* gene encodes a cytosolic zinc-binding enzyme that catalyzes the hydrolysis of N-acetylated amino acids to acetate and free aliphatic amino acids (Sommer et al., 2011).

Here, we report an infant with neurological findings such as seizures, neurodevelopmental delay and hypotonia. WES analysis of the proband and both parents identified the novel *de novo ATP7A* variant c.3642_3649dup (p.Ala1217Aspfs*2), leading to a diagnosis of Menkes disease, and the co-occurrence of homozygous variant c.1057C>T (p.Arg353Cys) in the *ACY1* gene causative of Aminoacylase-1 deficiency. Thus, the novel *ATP7A* mutation associated with Menkes disease expands the *ATP7A* gene spectrum.

## Case presentation

### Clinical features

**FIGURE 1:**
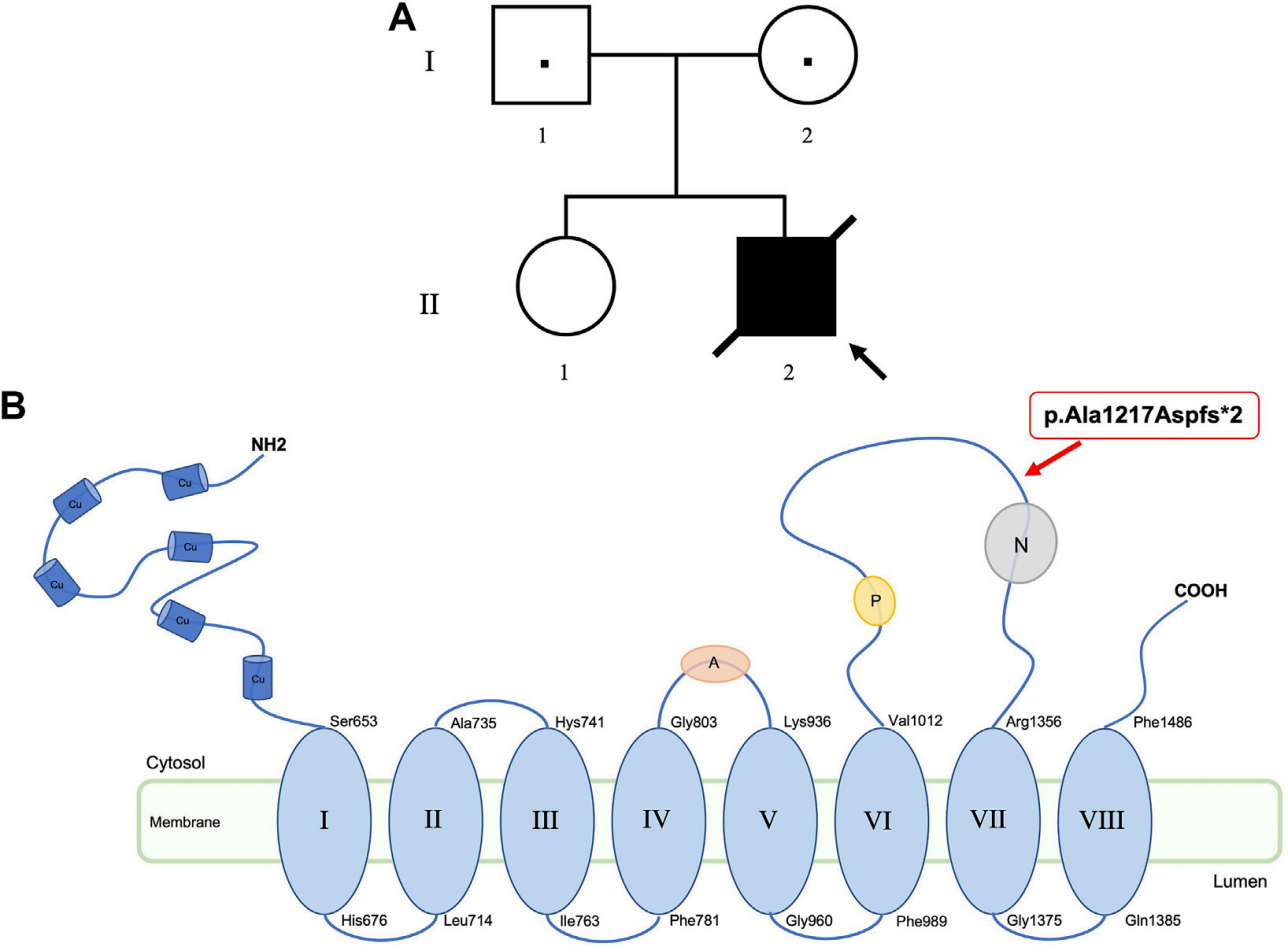
Pedigree of the family **(A)**. Location of *ATP7A* variant (p.Ala1217Aspfs*2) at protein level. ATP7A has six copper binding sites (Cu), eight transmembrane domains, an activation domain (A), a phosphorylation domain (P), and a nucleotide domain (N) **(B)**.

The proband II-2 (Figure 1A) was the second-born of non-consanguineous healthy parents (I-1 and I-2). The elder sister (II-1) was referred also healthy. The family history was negative for genetic conditions.

The patient was born at 36 + 4 gestational weeks, after normal pregnancy and uncomplicated delivery. At birth, the child was 2680 g (75th centile) and newborn screening at the age of 6 days showed increased trypsin levels (66.5 ng/mL; cut-off 60 ng/mL).

In the perinatal period he presented episodes of asymptomatic hypoglycemia and neonatal jaundice resolved after a normal course of phototherapy.

In the first weeks of life recurrent short episodes of tremor during sleep, mainly in the upper limbs, was reported, which slowly reduced over following months. Sleep-wake rhythm was regular, with occasional sudden awakening with intense crying. Growth was regular, he received mixed breastfeeding.

At 4 months of age, the child was 5000 g (<third centile), 58 cm long (<third centile), presented an occipitofrontal circumference of 40,5 cm (15th centile). The proband began to have sudden and recurrent episodes of staring with hypomotility, lasting few seconds to minutes, with a rapidly progressive course up to episodes of psychomotor arrest with eyes wide open and unresponsiveness. Then followed by parent’s description of tonic-clonic limb movements and spasms. Because of these episodes the patient has been transferred to our hospital.

Genetic evaluation (age 4 months) showed plagiocephaly, round face, sparse, twisted and lightly pigmented hair, short palpebral fissures, small nose, sagging cheeks, bilateral ear lobes crease. Hands with simplified dermatoglyphics.

Neurological evaluation showed hyporeactivity to external stimuli with irritability, severe plagiocephaly, strabismus without evocable eye fixation and with abnormal ocular movements (roving, nystagmus), axial hypotonia, reduced spontaneous limb movements with normal stretch reflexes.

Routine blood and cerebrospinal fluid (CSF) analysis, screening and cultural examinations for infectious diseases (HSV1-2-6, VZV, CMV, enterovirus, RSV, SARS-CoV-2) were negative. Routine chest X-ray and abdominal ultrasound were unremarkable, except for a pseudo diverticular aspect of the bladder walls with possible urethral dilation.

Brain ultrasound was normal, while electroencephalogram (EEG) revealed asymmetric activity with persistent paroxysms of delta slow wave with superimposed spikes over left parieto-occipital region.

A prolonged EEG/aEEG (amplitude-integrated EEG) recording in PICU showed moreover several discharges of high amplitude spike-slow wave complexes over left centro-temporal region, lasting 30–90 s, with no clinical expression or with occasional correlation with perioral myoclonia. Clinical seizures with staring, lateral eye deviation, upper limbs spasms and oral automatisms were also recorded.

In the acute phase seizures were treated with intravenous midazolam, with only temporary effect. Then intravenous phenobarbital achieved a progressive reduction of EEG discharges and a good seizure control.

**FIGURE 2:**
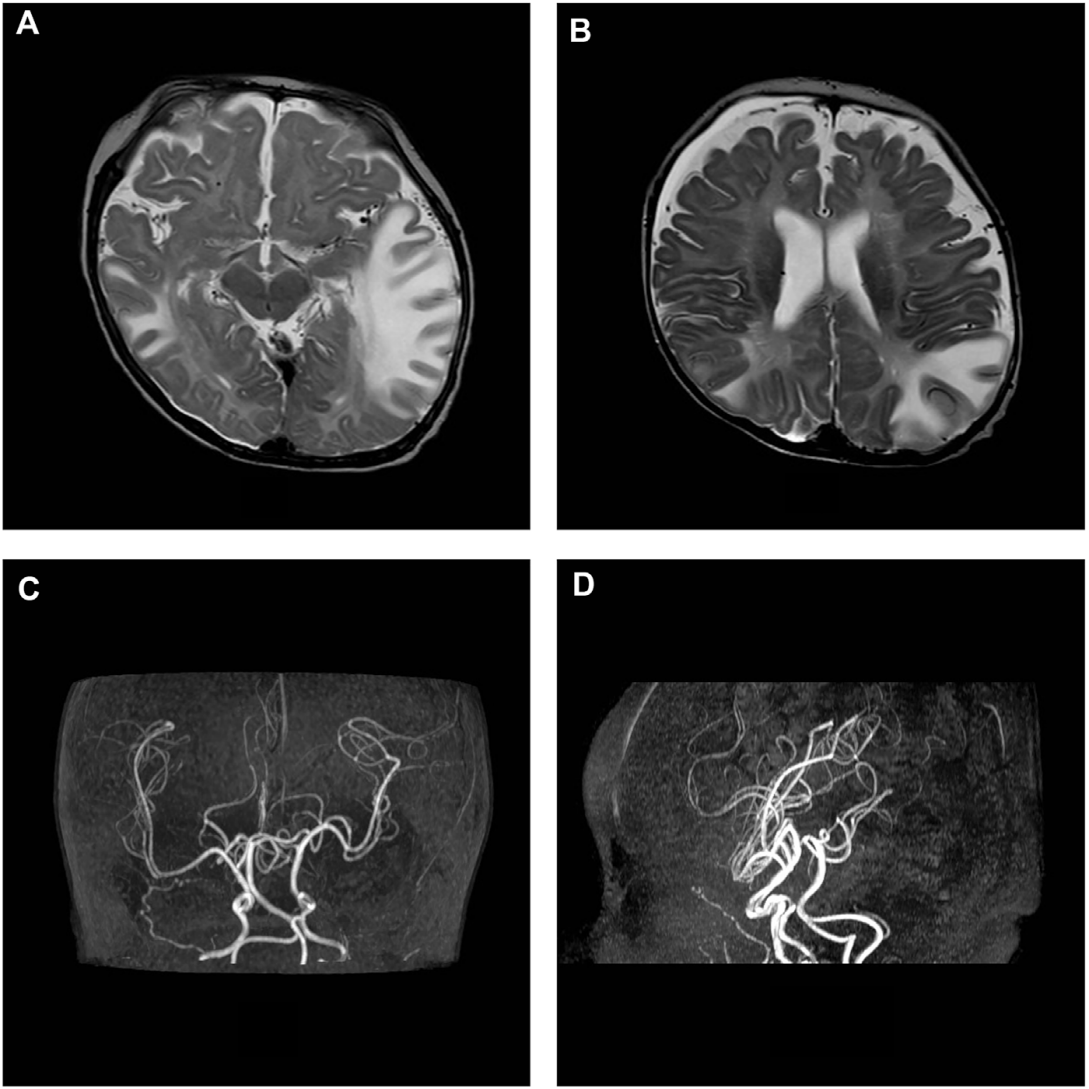
MRI axial T2-w images show asymmetric tumefactive lesions of the temporal and parietal white matter, increase in size of subarachnoid spaces and subtle frontal bilateral subdural collections **(A, B)**. On AngioRM sequence intracranial arteries tortuosity is evident **(C, D)**.

Brain MRI, performed at 5 months of age, showed increase in size of subarachnoid cerebellar and cerebral spaces and ventricular system; subtle frontal bilateral subdural collections, extensive asymmetric tumefactive lesions of the temporal, parietal and occipital white matter, swelling and T2 weighted images hyperintensity of basal ganglia bilaterally, increase of perivascular spaces with cystic appearance in frontal lobes, abnormal myelination of white matter and thinned corpus callosum (Figures 2A,B). On AngioRM sequence marked intracranial artery tortuosity was evident (Figures 2C,D).

Clinical evolution showed seizures’ relapse at 6 months, which became progressively more frequent and pharmacoresistant. Subsequent antiepileptic treatment with phenytoin, vigabatrin, sodium valproate, clonazepam obtained only partial and temporary seizure control.

No psychomotor or postural acquisition has been achieved in the following months. While progressively increased limbs muscular tone with axial hypotonia, hyperreflexia, and hyperkinetic/dyskinetic movements became evident.

MRI control at 8 months of age revealed atrophic evolution of tumefactive lesions with persistence of signal alteration in the right temporal lobe and basal ganglia were detected. Subarachnoid spaces, ventricular system and subdural collections were increased. White matter myelination did not progress. Intracranial vessels tortuosity was confirmed.

Moreover, patient suffered from frequent respiratory infections, complicated by poor secretion management and episodes of aspiration pneumonia. Thus, developing pulmonary atelectasis and respiratory failure. He then died at 12.5 months of age.

### Metabolic screening

**FIGURE 3:**
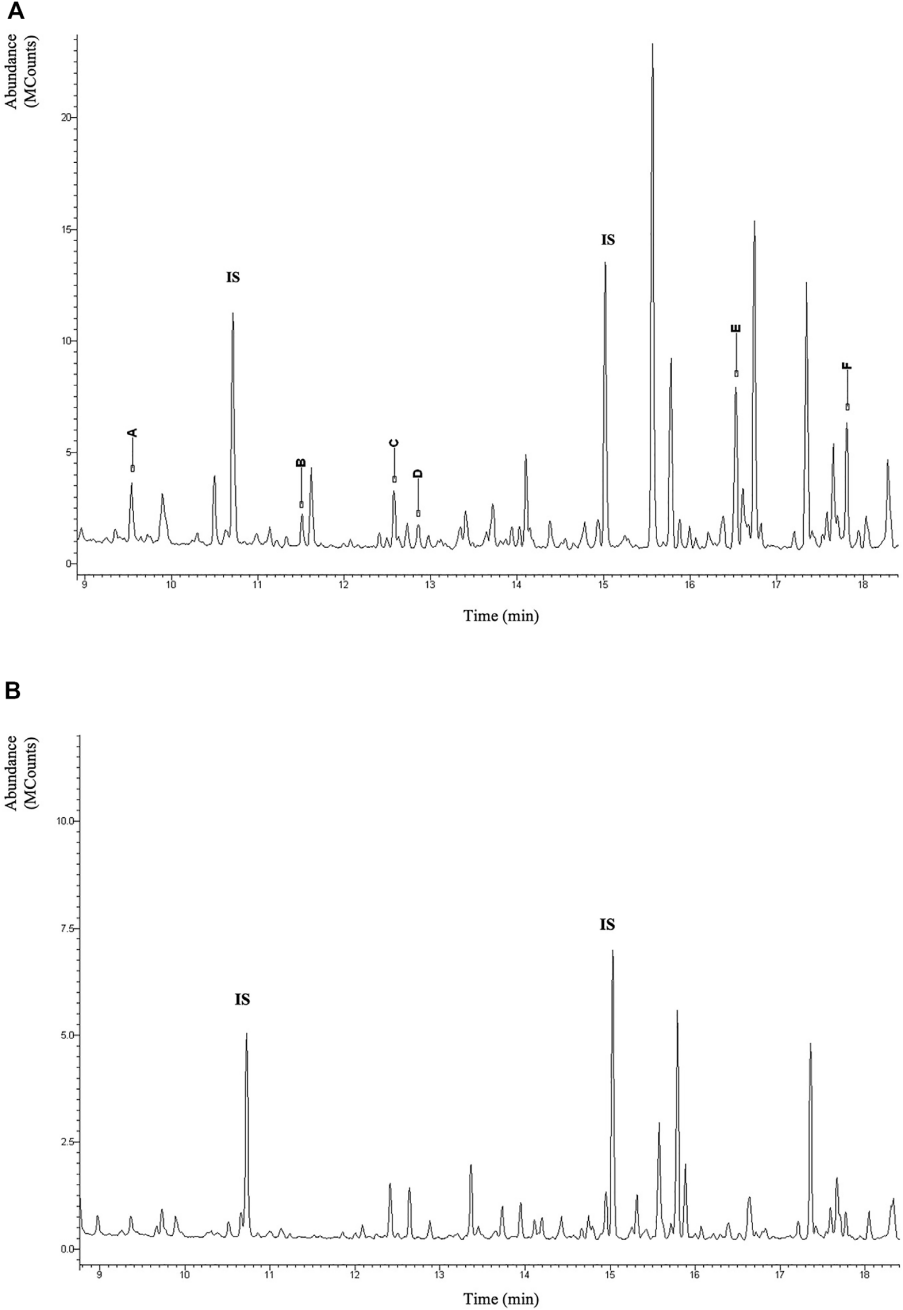
Section of the total-ion chromatogram of urinary organic acid of the patient after extraction with ethylacetate and derivatization with BSTFA + TMCS (1%) at 60°C for 30 min. IS (Internal Standard). In the figure the TMS derivatives of the N-acetylated amino acids are highlighted with capital letters: (A) N-acetyl-alanine; (B) N-acetyl-glycine; (C) N-acetyl-leucine; (D) N-acetyl-isoleucine; (E) N-acetyl-methionine; (F) N-acetyl-glutamate **(A)**. Normal pattern **(B)**.

Urinary organic acids analysis by gas chromatography-mass spectrometry (GC-MS) revealed N-acetylated derivatives of methionine, glutamic acid, alanine, glycine leucine, valine, and isoleucine in large amounts. This pattern in Figure 3A is considered characteristic for ACY1 deficiency. In contrast, urinary organic acids of the sister and parents were unremarkable.

In the interim, serum ceruloplasmin (0.04 g/L; normal range, 0.2–0.6 g/L) as well as his serum copper (<60 ug/dL; normal range, 80–150 ug/dL) was noted to be low, whereas lactate levels were mildly elevated (values ranging from 2.4 to 5.4 mmol/L; normal range, 0.5–2.2 mmol/L). These metabolic and clinical findings were highly suggestive for Menkes disease.

### Genetic analysis

Karyotype, aCGH, and methylation analysis of the 15q11.2-q13 locus did not identify any significant alteration. The proband (at 4 months of age) and both parents underwent WES analysis on DNA extracted from peripheral blood leukocyte as previously reported (Pezzani et al., 2018). According to the pedigree and the clinical features, the analysis allowed the identification of two different variants, each associated with the two supposed diseases, establishing the diagnosis of a compound phenotype.

The pathogenic homozygous missense variant c.1057C>T (p.Arg353Cys) in exon 14 of the *ACY1* gene (3p21.2; #104620) was identified in the proband and was inherited from both heterozygous parents. The variant, already reported in dbSNP (rs121912698), has a low frequency in general population (ƒ = 0.00252) according to gnomAD Genomes database v.3.1.1 and was predicted to be damaging by several prediction tools (Mutation Taster, PROVEAN, LRT, fathmm_MKL). According to ACMG criteria, the reported variant is classified as pathogenic, and it has been described by Van Coster et al. (2005) (Van Coster et al., 2005) in an individual affected with Aminoacylase-1 deficiency. Notably, this variant is annotated in ClinVar with conflicting interpretations of pathogenicity (RCV000514755): some laboratories labeled c.1057C>T in *ACY1* as pathogenic, in contrast others reported it as a variant of uncertain significance. Although this variant appears to lead to a reduction in ACY1 enzyme activity, it is unclear if ACY1D is the cause of reported neurological phenotypes, so the clinical pathogenicity of this variant remains elusive and uncertain.

The second identified variant was a novel hemizygous *de novo* frameshift c.3642_3649dup (p.Ala1217Aspfs*2) in the *ATP7A* gene (Xq21.1; #300011). The variant, shown in Figure 1B at protein level, introduces a premature termination codon, thus resulting in a truncated protein or in the complete absence of the protein, due to the non-sense-mediated decay predicted by MutationTaster tool. The detected variant, not reported in dbSNP or in ExAC, 1000G or gnomAD databases, is classified as pathogenic according to ACMG criteria. Moreover, the frameshift mutation was confirmed to be a novel variant after comparison with the Human Gene Mutation Database (www.hgmd.org) and the *ATP7A* database in the Leiden Open Variation Database 3.0 (www.LOVD.nl/ATP7A).

## Discussion and conclusion

Trio WES analysis according to the pedigree and the proband clinical features identified the novel *de novo* variant c.3642_3649dup (p.Ala1217Aspfs*2) in the *ATP7A* gene, leading to a diagnosis of Menkes disease, and concurrently, the homozygous pathogenetic *ACY1* variant c.1057C>T (p.Arg353Cys), inherited from both healthy heterozygous parents and causative of Aminoacylase-1 deficiency.

The neonatal clinical features of Menkes disease are non-specific (e.g., prolonged jaundice, hypoglycemia) and usually not sufficient to suspect this rare condition in the first instance. For this reason, diagnosis is often delayed until around 6–12 weeks of age or beyond, when more overt symptoms such as failure to thrive, hypotonia, and seizures present (Kaler et al., 2003). Moreover, the presence and partial overlapping clinical presentation of the two distinct conditions (Menkes disease and Aminoacylase-1 deficiency) in our patient may have contributed to a complexity of diagnosis of Menkes disease. Any possible clinical manifestations attributable to ACY1 deficiency were mostly “included” among those manifested by Menkes disease (hypotonia, neuromotor delay/arrest, epilepsy). The two pathologies provided may have provided a “contribution” to the severity of the clinical picture, but from an exclusively clinical and also radiological point of view it was not possible to discriminate between them. To appreciate the overlap and differences about both conditions, an overview of the symptoms in the proband and the known phenotype spectrum of diseases are shown in Table 1.

**TABLE 1:** Overview of the symptoms in the proband and the most commonly reported phenotypes of both conditions.

Clinical features	Menkes disease	ACY1 deficiency
**Hypotonia**	✓	✓
**Hypertonia**		✓
**Loss of developmental milestones**	✓	
**Failure to thrive**	✓	
**Intellectual disability**		✓
**Language delay**		✓
**Seizures**	✓	✓
**Behaviour abnormalities**		✓
**Sensorineural hearing deficit**		✓
**Abnormal hair**	✓	
**Vascular tortuosity**	✓	
**Bladder diverticula**	✓	
**Gastric polyps**	✓	
**Bone abnormalities**	✓	
**Premature death**	✓	

Aminoacylase-1 deficiency has been associated with a wide variety of clinical presentations that are in stark contrast to Menkes disease. Notably, most patients with diagnosis of Menkes disease have the severe form, including our infant who followed the expected natural history with limited life span (Kaler et al., 2008). The high mortality rate and neurocognitive decline are due to both low copper levels and various copper-dependent enzymes as a cofactor (such as cytochrome c oxidase, lysyl oxidase, dopamine ß‐hydroxylase, peptidylglycine α‐amidating monooxygenase, superoxide dismutase, and tyrosinase) (Kaler, 2011).

To date, copper histidinate injections are the most promising treatment for Menkes disease, understanding of the underlying pathophysiology of the disease would also help develop new therapeutic strategies. Many studies have shown some benefit in delaying the progression of neurocognitive decline with subcutaneous copper histidine therapy, especially when administered early in life (Kim et al., 2015; Vairo et al., 2019). For instance, a patient with a non-sense *ATP7A* mutation (Arg201*) received copper histidinate injections starting at 8 days of life and had an excellent long‐term therapeutic response with no neurocognitive deficits (Kaler et al., 2009). The same variant in an infant with delayed Menkes diagnosis (Woodfin et al., 2019), and then copper histidinate treatment, led to premature death, underlying the need of early diagnosis. Therefore, copper histidinate therapy might only modify the progression of disease if initiated within 30 days after birth, as has been reported elsewhere (Vairo et al., 2019; Fujisawa et al., 2022). In our case Menkes disease was diagnosed at 4 months of age, thus the patient was treated with symptomatic management because copper histidine treatment would not have been effective.

In conclusion, we suspect that the poor outcomes of our patient were related to both delayed copper therapy and severe *ATP7A* mutation, in addition to the concurrent presence of ACY1 deficiency. This highlights the importance of immediate diagnosis, and the need of efficient newborn screening methods for prompt treatment, as well as the utility of WES as first-tier genomic test for phenotypes with a suspected genetic etiology. Compared to traditional genetic testing (Karyotype, aCGH) and multigene panels sequencing, WES-based diagnosis may represent a powerful agnostic tool to solve complex cases such as ours, avoiding missing a second diagnosis and delaying any possible treatments, and allowing an efficient and rapid identification of the genetic cause in rare and ultra-rare conditions (Mergnac et al., 2022; Moresco et al., 2022).

## Data Availability

The raw data supporting the conclusions of this article will be made available by the authors, without undue reservation.
